# The effects of stocking density on behavior and biological functioning of penned sheep under continuous heat load conditions

**DOI:** 10.1093/jas/skad223

**Published:** 2023-07-01

**Authors:** Bonnie T Mayes, Peta S Taylor, Frances C Cowley, John B Gaughan, John M Morton, Brendan P Doyle, L Amy Tait

**Affiliations:** School of Environmental and Rural Science, University of New England, Armidale, NSW 2351, Australia; School of Environmental and Rural Science, University of New England, Armidale, NSW 2351, Australia; School of Environmental and Rural Science, University of New England, Armidale, NSW 2351, Australia; School of Agriculture and Food Sciences, The University of Queensland, Gatton, QLD, 4343, Australia; Jemora, East Geelong, VIC, 3219, Australia; School of Environmental and Rural Science, University of New England, Armidale, NSW 2351, Australia; School of Environmental and Rural Science, University of New England, Armidale, NSW 2351, Australia

**Keywords:** allometry, heat stress, live export, sheep welfare

## Abstract

Stocking density may impact sheep welfare during live export voyages that occur under hot and humid conditions. The aim of this study was to assess the welfare implications for sheep housed at three allometric stocking densities (*k* = 0.030, 0.033, 0.042), while exposed to hot and humid climatic conditions. For 21 d, Merino wethers (*n* = 216) were housed in 12 pens of 18 wethers, in two climate-controlled rooms where wet-bulb temperature (*T*_WB_) mimicked the conditions of a live export voyage with high heat and humidity, and limited diurnal variation. Scan sampling of standing and lying behaviors was conducted on days 2, 5, 8, 11, 15, 18, and 20, at hourly intervals. Agonistic interactions were scored continuously on the same days between 1750 and 1800 h. Liveweights were recorded at the start and end of the study. For a subset of focal wethers (3 per pen), whole blood variables were assessed at the start and end of the experiment, along with fecal glucocorticoid metabolites (FGCM), which were also assessed on days 7 and 14. Rumen temperatures (*T*_RUM_) of focal wethers were recorded at 10-min intervals, and their respiration rates (RR) were measured every 2 h on days 1, 3, and from days 7 to 21. Focal wethers were slaughtered for necropsy after the study, and both adrenal glands were excised and weighed. The expression of some lying positions was impaired at high stocking densities, and lying with outstretched legs increased at high *T*_WB_. For respiration rates, there was an interaction between stocking density and *T*_WB_, such that RR was reduced by the provision of additional space at high *T*_WB_. *T*_RUM_ was relatively unaffected by stocking density but increased at higher *T*_WB_, and any effects of stocking density on FGCM concentrations, liveweights (LW), adrenal gland weights or blood variables were minimal. Necropsy examination showed no indication that the wethers had experienced ongoing respiratory distress. These results suggest that the wethers were able to cope with these increases in stocking density under the conditions imposed. However, based on this evidence, the provision of additional space under hot conditions may be beneficial to facilitating the expression of some lying positions. Whilst the experiment was designed to emulate certain conditions relevant during live export voyages, other factors that may induce stress during this mode of transport were not present, and so the conclusions must be interpreted in the context of the experimental conditions.

## Teaser

For sheep housed intensively under hot and humid climatic conditions, stocking density impacted lying behaviors, but had limited important effects on heat stress and physiology in the highly controlled experimental environment.

## Introduction

Australia is one of the largest exporters of live animals (Aguilar et al., 2020) and nearly half a million sheep were exported from Australia in 2021 (LiveCorp, 2022). For voyages traveling to the Middle East and departing Australia during winter, there is a rapid transition to warm climatic conditions as ships cross the equator and approach destination ports (Carnovale and Phillips, 2020). Several heat stress-related mortality incidents have occurred on voyages to the Middle East (McCarthy, 2018) and stress associated with exposure to high temperatures has become an important concern for community and industry (Dunston-Clarke et al., 2020).

Wet-bulb temperature (*T*_WB_), a measure determined jointly by air temperature and relative humidity, is used within the live export industry to assess heat stress risk during voyages (HSRA Technical Reference Panel, 2019). Wet-bulb temperatures above 30 °C are considered a heat stress risk for sheep (Jubb and Perkins, 2019), and during voyages, *T*_WB_ can reach up to 34 °C (Stockman et al., 2011). The temperature humidity index (THI) is another common indicator of heat stress risk in animals (Srikandakumar et al., 2003), and is jointly determined by air temperature and relative humidity. Using the THI equation of Marai et al. (2007), it has been suggested that values less than 24.3 indicate the absence of heat stress risk, values between 24.3 and 25.6 indicate moderate heat stress risk, values up to 32.3 indicate severe heat stress risk, and values beyond this range indicate extreme heat stress risk (Lees et al., 2017).

The effects of high T_WB_ and THI are exacerbated by the limited diurnal variation (i.e. difference in the daily maximum and minimum) in temperature during live export voyages, caused by the limited reduction in temperature at night in equatorial regions (Beatty, 2005; Phillips, 2016). This results in sheep accumulating additional heat during the night, when they would usually have the opportunity to dissipate excess heat (Stockman et al., 2011; HSRA Technical Reference Panel, 2019). Since 2018, government and industry efforts have partially mitigated heat stress risks for sheep by implementing a voluntary moratorium on sheep exports to northern hemisphere destinations during the northern summer (Petrie, 2019). This initiative is now supported by a legislative ban on sheep voyages departing Australia between 1 June and 14 September (Department of Agriculture Water and the Environment, 2021b). Voyages departing during the month of May, and between the September 15 and 31 October (defined as the ‘shoulder period’ of the northern hemisphere summer), are subjected to legislative adjustments in stocking densities to mitigate heat stress. High stocking densities, with animals in close physical contact with each other, lead to reduced body surface area through which heat can be dissipated, restricted airflow around sheep, and increased heat exchange between individuals (Knowles et al., 1998; MAMIC, 2001; HSRA Technical Reference Panel, 2019). Since 2018, stocking densities during sea voyages have been determined using allometric principles (Australian Livestock Exporters’ Council, 2018). The allometric equation used to estimate spatial requirements (Petherick and Phillips, 2009) is


$$\text{Area per sheep }({\text{m}}^{2})=k{\text{W}}^{2/3},$$

where *W* is the liveweight of animals (W) in kg and *k* represents a space allowance coefficient constant. Voyages departing Australia are stocked at a *k* value of 0.030 in normal circumstances, and a higher *k* value of 0.033 (i.e., more space) is applied for voyages that depart during the shoulder period (Department of Agriculture Water and the Environment, 2021a). Based on published research and voyage footage available at the time, an independent review of on-board conditions during voyages identified a *k* value of 0.033 as suitable for shoulder period voyages (McCarthy, 2018). However, this recommendation was based on reported negative effects for animals stocked at *k* values less than 0.033 under *thermoneutral conditions* (Petherick and Phillips, 2009), with no specific regard for how stocking density may interact with hot climatic conditions.

To date, limited research has been conducted to determine effects of stocking densities for sheep exposed to high heat loads. Some voyage-based stocking density research during hot conditions has been conducted, but the researchers were limited in what assessments could be implemented during a voyage and made conclusions based on time spent lying and final liveweight (LW) alone (Ferguson and Lea, 2013). In addition, treatment stocking densities were also confounded with changes in group size, limiting the accuracy of any conclusion that can be made. As such, the effect of stocking density on sheep welfare under high heat loads during transport persists as a gap in the current literature (Nielsen et al., 2022), and more evidence is required to define thresholds in stocking density adjustments that can safeguard the welfare of sheep being exported during the shoulder period of the northern hemisphere summer.

The aim of this study was to assess the welfare implications for sheep housed at three stocking densities and exposed to shoulder period climatic conditions. We anticipated that reducing space allowance would lead to reduced sheep welfare, as indicated by various physiological and behavioral assessments, and that the impact of reduced space would be exacerbated at higher wet-bulb temperatures with limited diurnal respite.

## Materials and Methods

The experiment was undertaken in the climate-controlled rooms at the Queensland Animal Science Precinct (QASP), Gatton, QLD, Australia. The conduct of the experiment was approved by The University of Queensland Animal Ethics Committee under the Animal Care and Protection Act, 2001 (approval ARA 2021/AE000088).

### Experimental design

The experiment tested three allometric stocking density *k* values; 0.030, 0.033, 0.042. Given the liveweights of the study animals, these *k* values provided approximately 0.34, 0.37, or 0.48 m^2^ of pen space per wether, respectively. Each treatment *k* value was replicated randomly among two blocks of three pens in each of two climate-controlled rooms so that each treatment was replicated twice in each room and four times in total. The experiment consisted of an 8 d adaptation period, followed by a 21 d exposure to relevant voyage climatic conditions and stocking densities; the 21 d experiment period is comparable to the length of live export voyages traveling from Australia to the Middle East (Caulfield et al., 2014).

### Animals and induction protocol

Two hundred and thirty-one 12 to 18 mo old Merino wethers (mean live weight ± SD: 39.7 ± 4.25 kg) were trucked to the experiment location from the New England region. Wethers had been inducted on their single farm of origin, two weeks prior to transport, during which each individual was shorn, weighed, identified using numbered ear tags, body condition scored, and given an oral anthelmintic (Startect, Zoetis Australia, Silverwater, NSW), external parasite pour-on treatment (Cydectin, Virbac Australia, Milperra, NSW), a 5-in-1 clostridial vaccination (Ultravac, Zoetis Australia, Silverwater, NSW) and scabby mouth vaccination (Scabigard, Zoetis Australia, Silverwater, NSW). After transport to the study site, the wethers were held in a large outdoor yard (36 x 12 m) for one night with access to *ad libitum* water and half their daily ration made up of 50 % oaten chaff and 50 % pellets based on 2.85 % of the average LW. The following day, the 8 d adaption period began, during which the wethers were introduced to a commercial shipper pellet diet with 9.5 MJ ME/kg DM and 12.1 % CP (Macco Feeds, Western Australia, Australia). Wethers were fed a mix of 50% oaten chaff and 50% pellets for day 1 of adaptation, based on a feeding rate of 2.85% of the average LW. The amount of oaten chaff was reduced to 25% and 15% of the diet for days 2 to 3, and 4 respectively. From day 5 of adaptation onwards, wethers were fed pellets exclusively to replicate voyage conditions. During the adaptation and experimental periods, the wethers were fed twice daily at 0900 and 1530 h.

### Treatment allocation and adaptation

Wethers were randomly allocated to 12 experimental groups of 18 wethers (*n* = 216 wethers), and 15 wethers were retained as spare wethers. In each experimental group of 18, three were randomly selected as focal wethers. The day after arrival at the study site, all wethers were drafted into four groups of 57 or 58 wethers, containing three experimental groups and three or four spare animals, and moved into four large pens (3.47 × 8.68 m) within the climate-controlled rooms to begin the adaptation period. Spare wethers were housed with experimental wethers during the adaptation period to adapt to the experimental facility, in case replacement of an experimental wether was required prior to the beginning of the experimental period. The wethers were housed at a *k* value of 0.044 in the adaptation pens, which permitted them more space than any of the treatment stocking densities. Each pen contained six stainless steel automatic water troughs and 14.6 cm per wether of linear feed trough space. Feed buckets were fastened to the outside of the pens so as to not take up usable space within the pens, and wethers put their head through a gap (approx. 40 cm high) in the rail to access feed.

For the duration of the adaptation and experimental period, plastic flooring panels (STEPPER XL; MIK International, Ransbach-Baumbach, Germany) designed for sheep and goats were laid over the top of the original metal grate flooring to improve the suitability of the flooring for sheep. Fecal matter and urine fell through these flooring pens to enter the draining system beneath the original grate. Wethers were exposed to artificial lighting between 0600 and 1800 h, which was set to fade in and out over a 2-min period at these times.

### Treatment pen design

On the afternoon of day 8 of adaptation, the four large adaption pens were each split into three treatment pens, by inserting additional panels. Experimental wethers were then individually drafted into their indoor treatment pens, and spare wethers were drafted into the outdoor spare pen. Once all 18 wethers were in a treatment pen, the panels were fastened to maintain allocated floor space within ± 0.001 m^2^ of the area required by the allometric equation, area per wether in m^2^ = *k*W^2/3^, where *k* was the treatment *k* value and *W* was the mean live weight (kg) of the 18 wethers in each pen, as measured on day 6 of the adaptation period. This area was then multiplied by 18 to correspond to the 18 wethers in the pen. Water and feed troughs were positioned so that when adaptation pens were divided, each treatment pen contained two water troughs in opposite corners and 14.6 cm per wether of feed trough space, along the outside of two opposite sides of each pen (Supplementary Figure S1).

### Animal management

During the adaptation and experimental periods, handlers entered the climate-controlled rooms twice per day (0730 h and 1430 h) for health and welfare checks, cleaning (including hosing out pens), and feeding. The health of all wethers was checked first, using the Gunson inspection method (Jubb and Perkins, 2019), and then water troughs were cleaned as required. The drains located under the pens were washed out using a high-pressure hose in each room; care was taken to not wet the wethers or concrete floors around the pens. Excess water in the walkways was scraped off into the drainage system. At the conclusion of hosing, all feed refusals and fecal material were removed from troughs. Pens were then fed in a random order. The process was the same in the afternoon as in the morning.

### Climatic conditions

**Figure 1.**
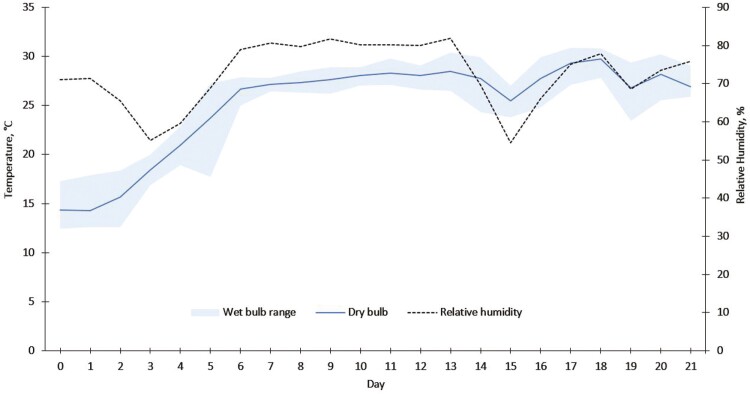
Daily means of dry-bulb temperature and relative humidity across both climate-controlled rooms for each of days 0 (the last day of the adaptation period) to 21 (the last day of the experimental period). Shaded zone indicates the minimum and maximum observed wet-bulb temperatures across both rooms on each day. The mean and maximum deviation from the desired wet-bulb temperature was 0.4 and 3.5 °C for room 1, and 0.2 and 3.3 °C for room 2.

Wethers spent the 8 d adaptation period housed at constant thermoneutral conditions (average *T*_WB_ throughout the adaptation period = 14.7 °C) in the climate-controlled rooms, based on expected average ambient conditions for the main port for Australian sheep exports, Fremantle (Western Australia, Australia), during the month of September. From the climate data, the THI was calculated according to the equation described by Marai et al. (2007): THI = *T*_A_ −(0.31 −(0.31 × RH/100) × (*T*_A_ – 14.4)). The THI throughout the adaptation period was 17.6. From d 0 (the eighth day of the adaptation period), the climatic conditions of both rooms were increased simultaneously to the experimental temperature and humidity protocol (Figure 1). The THI for each day was also calculated (Supplementary Figure S2). This protocol adjusted the dry-bulb temperature and relative humidity hourly, following observed climate data collected from a live export voyage from Fremantle, Western Australia, to Kuwait in September 2019.

Two Kestrel D3 Fire Drops (Nielsen-Kellerman, Pennsylvania, USA) continuously logged data for temperature and humidity in each room for the duration of the experiment, at 10-min intervals. One logger was hung at sheep head height from the ceiling on either side of each of the two rooms in a cavity between two treatment pens. Four fans mounted in the top corners of each room were set to the highest velocity and to oscillate for the entirety of the adaptation and experimental periods; fans facilitated general air circulation but were not directed towards the wethers or the Kestrel Drops. Handheld ToxiRAE Pro PID ammonia monitors (Honeywell, North Carolina, USA) were used to record the ammonia concentration of each room twice a day (mean ± SD 21.1 ± 8.4 ppm) before hosing occurred, to ensure that concentrations were not reaching limits that may impair sheep or human health (≥ 25 ppm; (Safe Work Australia)). Air filters at the back of each climate-controlled room were replaced every 3 d to maintain a wind speed at the air extraction point greater than 1.4 m/s.

### Behavior assessments

Video footage was recorded by fixed infra-red cameras (MR6822E2 and LR832, Lilin Australia Pty Ltd, Lidcombe NSW) positioned above each individual experimental pen for the entire duration of the 21 d experimental period. Cameras were positioned so that the entire pen and all animals were visible throughout the study. Behaviors were assessed after the experimental period by one experienced observer, using a scan-sampling approach for standing and lying behaviors, and a continuous observation approach for agonistic social interactions. All behavior data were collected in Microsoft Excel (2016).

All wethers in each pen had their standing or lying position recorded (Tables 1 and 2, respectively) in a scan-sampling approach, at hourly intervals (using still images) for a 24-h period on days 2, 5, 8, 11, 15, 18, and 20. Six time points were excluded on each day, when the wethers were disturbed due to human presence (0700, 0800, 0900, 1000, 1400, and 1500 h).

**Table 1.** Ethogram utilized to record standing behaviors for all wethers at scan sampling observation times. Standing positions are mutually exclusive.

Standing position	Definition
Standing	Wether is upright with at least three hooves in contact with the pen floor
Drinking	Wether is standing and head is extended outside of the pen with muzzle lowered into nosebowl trough so that tip of muzzle is not visible
Feeding	Wether is standing and head is extended outside of the pen with muzzle lowered into feed trough so that tip of muzzle is not visible
Standing with head out of pen	Wether is standing with head extended outside of the pen with both ears are through the rail but the muzzle is not lowered into feed or water troughs
Standing with head up	Wether is standing and has head inside the pen and elevated above the height of the shoulder
Standing with head down	Standing wether has head inside the pen and in line with, or below, the height of the shoulder
Lying	Wether is lying in accordance with any description under “position of legs” in Table 2.

**Table 2.** Ethogram utilized to record lying behaviors for all lying wethers at scan sampling observation times. Positions within each category are mutually exclusive. Each category was assessed for each lying wether.

Category of lying behavior	Lying position	Definition
Position of legs	Position 0	Wether is lying with all four legs kept close to body. There is no gap between the lower portion of a back leg and the body. No front legs are outstretched
	Position 1	Wether is lying with one leg outstretched from body. There is a visible gap between the body and the lower portion of the outstretched leg
	Position 2	Wether is lying with two legs outstretched from body. There is a visible gap between the body and the lower portion of the outstretched legs
	Position 3	Wether is lying with three legs outstretched from body. There is a visible gap between the body and the lower portion of the outstretched legs
	Position 4	Wether is lying with four legs outstretched from body. There is a visible gap between the body and the lower portion of the outstretched legs
	Unknown	Unable to see enough legs to determine position
Body contact	In contact with conspecific	Wether is lying with body or limb in direct contact with a conspecific
	No contact with conspecific	Wether is lying and is not touching a conspecific with either body or limb
	Unknown	Wether is lying with but cannot determine if wether is touching a conspecific or not
Head position	Head up	Wether is lying with head held up
	Head down	Wether is lying with head down placed on the floor or the pen, on themselves, or on a conspecific
	Head resting on conspecific	Wether is lying with head resting on a conspecific
	Unknown	Wether is lying but cannot determine the head position

Intra-observer reliability was assessed at the end of the data collection period by reanalyzing 34 images that had been analyzed at the start, middle, and end of the data collection period. Lin’s concordance correlation coefficients and intraclass correlation were calculated to indicate the level of agreement for all known standing and lying behaviors. The agreement was not assessed for lying four because of the rarity of this behavior in the test data.

Continuous behavior observations were conducted for all wethers in each pen for one 10-min period on each of days 2, 5, 8, 11, 15, 18, and 20, from 1750 to 1800 h. Hourly scan sampling observations indicated that at this time period, numbers of wethers lying were typically low, and so this observation period was chosen to represent a time at which wethers were standing and active, to detect a reasonable number of agonistic interactions. Agonistic interaction events were the only behavior counted and were defined as occurring when a wether made direct contact with a conspecific with head-butting, mounting, or pawing behaviors.

### Heat stress assessments

On day −2 (i.e., the sixth day of adaptation), rumen boluses (SmartStock, OK, USA) were orally administered to the 36 focal wethers, using a purpose-built applicator. Boluses were cylindrical (2.0 cm diameter × 7.0 cm length) and weighed 78 g. Before administration to wethers, each was tested for temperature stability and accuracy in a 40 °C water bath for 12-h. Any differences in recorded temperature during the water bath test were used to correct experimental data recorded by the boluses. Temperature data from boluses was transmitted to a base station outside the climate-controlled rooms via an antenna. This data was then transcribed to SmartStock (OK, USA) software so that temperatures were transmitted and recorded in Microsoft Excel at 10-min intervals, and then averaged for each hour for each focal wether over the 21 d period for analysis.

Respiration rates of each focal wether were assessed, from video footage at two-hourly intervals on days 1, 3, and 7 to –21. RR was calculated by counting the number of breaths in 15-s and then multiplying this value by four to convert the measure to breaths per min (bpm).

### Physiological sampling

**Figure 2.**
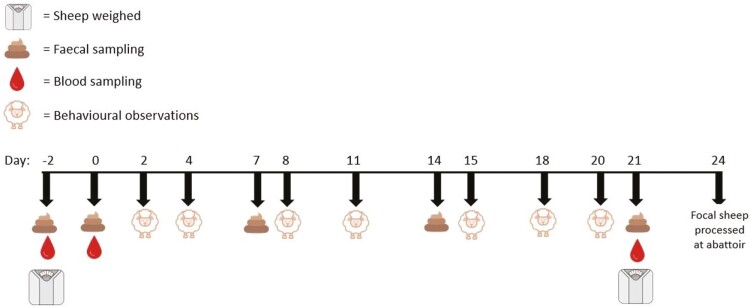
Study timeline depicting the days that liveweights, blood, feces, scan sampling behaviors, and agonistic behaviors were sampled. The 8-d adaptation period ended on day 0 and the experimental period was from days 1 to 21.

All wethers were weighed on day −2 (i.e., the sixth day of the adaptation period) and day 21; at each weighing, wethers had not been fed within the previous 12 h. Physiological sampling of focal wethers was performed between 0600 and 0700 h on sampling days before feed was provided. Baseline blood samples were collected from focal wethers on days −2 and 0 (i.e., the sixth and eighth day of the adaptation period), and on day 21 of the experimental period, to measure whole blood variables (Figure 2). Blood samples were taken via jugular venipuncture and collected into 10 mL K_2_-EDTA vacutainer tubes (BD, Lane Cove, NSW, Australia). Blood samples were stored at 4 °C for approximately 1-h post collection, then analyzed on a Sysmex XN-1000 (Sysmex, Macquarie Park, Australia) hematology analyzer and manual cell differentiation was performed.

Fecal samples were collected from focal wethers on days −2, 0 (baseline samples), 7, 14, and 21 (experimental period samples; Figure 2). A minimum of six fecal pellets were taken directly from the rectal ampulla of each focal wether before being immediately placed in a sample pot on ice. Fecal samples were frozen within 40-min after collection and stored at -20 °C until processing. Samples were oven-dried at 60 °C for 48-h and finely ground using a ceramic hand grinder. The individual samples were then analyzed using a method previously established for sheep (Mayes et al., 2022). Briefly, 100 mg of dried sample were reconstituted in 300 µL of double distilled water followed by mixing with a vortex shaker for 5-min. This was added to 2700 mL of 100% ethanol, vortexed for 10-min, then spun at 2000 × *g* for 10-min; the supernatant was decanted into glass tubes. Pellets were extracted again with 3 mL of 100% ethanol, spun at 2000 × *g* for 10-min and the supernatant added to the previous extract. The extracts were dried under airflow for 5 to 6-h and then were reconstituted in 500 µL of phosphate buffer saline (pH 7.4), vortexed for 10-min and spun at 1000 × *g* for 2-min. Concentrations of fecal glucocorticoid metabolites (FGCM) in the extract were measured in duplicate using the MP Biomedical I125 RIA cortisol Kit (# 07-221106, MP Biomedicals Australia, Seven Hills, NSW). The limit of detection was 2 ng/mL and the mean inter-assay coefficients of variation were 2.5% (11.5 ng/mL) and 9.5% (48.9 ng/mL). Results are expressed as ng of FGCM/g of dry feces.

On day 23, the 36 focal wethers were loaded onto a truck and transported for 2-h to a local abattoir (Highchester Meats Pty Ltd, Gleneagle, QLD, Australia), before being processed at 0630 h on day 24. With the assistance of an accredited veterinarian, a necropsy was performed on the 36 focal wethers and the condition of their diaphragm, heart, lungs, and liver was recorded. Then, both adrenal glands were excised, adjacent adipose tissue was removed and both adrenal glands were weighed to three decimal places.

### Statistical analyses

Statistical analyses were performed in Stata (StataCorp, Release 16. College Station, TX). *k* value, *T*_WB_ (when modeling outcome variables measured on multiple days), and room were simultaneously fitted as fixed effects in regression models. *k* value was fitted as a continuous variable unless otherwise specified, and hourly average *T*_WB_ for the room was fitted as a continuous variable in the models for which it was included. Room was fitted as a categorical variable. Linearity of relationships was assessed using fractional polynomial regression. For all outcome variables, there was no strong evidence for non-linear relationships for either *k* value or *T*_WB_ so any relationships between these and the outcome variables were assumed to be linear. Interactions between *k* value and *T*_WB_ (where fitted) were assessed. Results for the interaction terms were only reported when the *P* value for the interaction was low. Effects were considered to occur based on joint consideration of our assessment of the prior evidence for the association in combination with the *P* value (Goodman, 1999).

Mixed effects linear regression was used to assess the effects of *k* value on day 21 LW, total adrenal gland weight, concentrations of whole blood variables, and the neutrophil to lymphocyte ratio (log_e_-transformed). The individual sheep was the unit of analysis, pen was fitted as a random effect, and the baseline value for each sheep was fitted as a covariate. A repeated measures mixed effects linear regression model was used for analyzing FGCM concentration over time, with time point within sheep as the unit of analysis, sheep fitted as a random effect, a first- to assess effects order autoregressive residual correlation matrix used, and time point number included as a fixed effect.

Mixed effects linear regression was used to assess the effects of *k* value and hourly average T_WB_ on hourly average rumen temperature (*T*_RUM_) and RR at the end of the hour. The individual sheep-timepoint was the unit of analysis, sheep was fitted as a random effect, and a first-order autoregressive residual correlation structure was used. Lagged temperature effects on these variables have been previously reported (Hahn and Mader, 1997) and were investigated here; *T*_WBs_ for each hour up to 48-h prior were all found to significantly predict *T*_RUM_ and RR. The average *T*_WB_ of the prior 6-h was included as a climatic variable in these models, based on biological reasoning supported by (Hahn and Mader, 1997). Hourly average *T*_WB_ for the same hour as the outcome variable was included as the second climate variable as an immediate causal effect was biologically plausible and this variable was always a predictor of the outcome variable value, irrespective of the other climate variable included in the model during the investigation. Diurnal patterns in *T*_RUM_ were evident, so trigonometric (sine and cosine) predictors (Cox, 2006) with one complete period per 24-h were also included to account for time of day effects. Variations in T_RUM_ between individual wethers were assessed by fitting the same model and estimating the random intercept variance and, in a separate model, the random slope for *T*_WB_ for the same hour as the outcome variable. To assess the shape of the relationship between *T*_WB_ and *T*_RUM_, a three-dimensional fractional polynomial regression was fitted for *T*_WB_ at the time of *T*_RUM_ sampling. Allowing up to three dimensions enabled the greatest chance of identifying any curvilinear relationship that may have existed.

Kappa coefficients for the synchronization of lying were calculated for each 24-h period for each pen on days 2, 5, 8, 11, 15, 18, and 20 (12 pens by 7 d = 84 kappa coefficients) using numbers of wethers lying at each of the hourly scan sampling time points taken at 17 time points within the 24-h periods, according to the methods outlined by Rook and Penning (1991). Briefly, the kappa coefficients of agreement were calculated based on the number of wethers lying at each time point, and the extent of synchronization at each time was determined by comparing the number of pairs where both wethers were lying to the total number of possible pairs (Rook and Penning, 1991). The kappa coefficient was 1.0 only if, at each time point within the 24-h period, all wethers had the same lying status, for example, at some time points, all wethers were lying and at other time points, all wethers were not lying. A kappa coefficient of 0.0 indicates that the number of wethers lying within time points in the 24-h period was no greater than that expected by chance. Kappa coefficients were analyzed using mixed-effects linear regression models with pen-day as the unit of analyses, with pen included as a random effect, and with a first-order autoregressive residual correlation structure.

For other scan sampling data, the unit of analysis was the hourly time point within pen. Diurnal patterns in proportions of groups that were standing were evident so trigonometric (sine and cosine) predictors (Cox, 2006) with one complete period per 24-h were included to account for the time of day effects for all scan sampling behavior models. Numbers of wethers standing at each hourly time point were analyzed with zero-inflated negative binomial regression with robust standard errors that accounted for clustering of observation period within pen. For numbers of other positional behaviors, mixed effects generalized linear models regression was used with pen included as a random effect. For all models, the number of wethers eligible to exhibit the behavior was included in the model to account for the amount of exposure from which the behaviors were observed. Thus, for analyses of numbers of wethers standing, the amount of exposure was the total number of wethers in the group at the hourly time point, and for analyses of number of lying wethers that were in body contact with another wether, the amount of exposure was the number of wethers in the group that was lying at the hourly time point. The same exposure was used for analyses of number of lying wethers with head down (head positions 2, 3, and 4 pooled; Table 2) and analyses of number of lying wethers with legs outstretched from their body (lying positions 1, 2, 3 and 4 pooled, or lying positions 2, 3 and 4 pooled; Table 2). For analyses of number of lying wethers placing their head on a conspecific (head position 4; Table 2), the amount of exposure was the number of wethers lying with their head down. For analysis of number of standing wethers with their head down (head down; Table 1), the amount of exposure was the total number of standing wethers. Exposure counts did not include wethers for whom the behavior could not be observed on the image.

Mixed effects linear regression was used to assess effects of *k* value and *T*_WB_ on the number of agonistic interactions observed during the continuous observation period on days 2, 5, 8, 11, 15, 18, and 20. Pen-day was the unit of analysis and pen was fitted as a random effect. For this outcome only, there was some evidence of a non-linear relationship between agonistic counts and *k* value so *k* value was fitted as a categorical variable, rather than as a continuous variable.

In interpreting our results, we implemented the principles listed in the American Statistical Association 2016 statement on statistical significance and *P* values (Wasserstein and Lazar, 2016) and the methods described by Rothman and Lash (2021). Thus, we have considered our prior views about the probability that the null hypothesis is the truth in the target population when interpreting *P* values, as recommended by Goodman (2001), and considered alternative hypotheses that, based on our confidence intervals, are not compatible with our data and, assuming no prior information about the magnitude of effect, are unlikely to be the true value. As such, our description of effects as “important” or otherwise is not based on *P* values, but rather on the confidence interval limits in the context of real implications for sheep.

## Results

### Standing and lying behaviors

For intra-observer reliability, Lin’s concordance correlation coefficients and intraclass correlation coefficients indicated an acceptable level of reliability over time (Martin and Bateson, 2007; Table 3).

**Table 3.** Intra-observer reliability of known scan sampling behaviors recorded by the observer for 34 time point images, in terms of Lin’s concordance coefficient (LCC) and intraclass correlation (ICC).

Behavior	Intra-observer reliability	
	LCC	ICC
Standing	0.999	0.999
Lying 0	0.797	0.768
Lying 1	0.904	0.874
Lying 2	0.888	0.870
Lying 3	0.679	0.823
Lying 4	–	–
Body 1	0.991	0.982
Body 2	0.672	0.673
Head 1	0.923	0.851
Head 2	0.765	0.755
Head 3	0.939	0.976
Head 4	0.932	0.888
Stand head up	0.977	0.971
Stand head down	0.922	0.879
Stand drink	0.872	0.939
Stand feed	0.791	0.923
Stand head out	0.696	0.777

Kappa values for the synchronicity of lying (calculated for each 24-h period for each group) ranged from 0.05 to 0.32 and the *P* value for the effect of *k* value on kappa was 0.080. The largest effects consistent with these results (based on 95 % CI intervals) were small and would be unlikely to have any biological importance (estimated difference in mean kappa for each 0.01 increase in *k* value = 0.02; 95 % CI −0.002 to 0.04). *T*_WB_ had a small effect on synchronicity of lying. The mean kappa for lying was estimated as reducing by a factor of 0.01 for every 1 °C increase in *T*_WB_ (95 % CI −0.01 to −0.008; *P* < 0.001).

**Figure 3.**
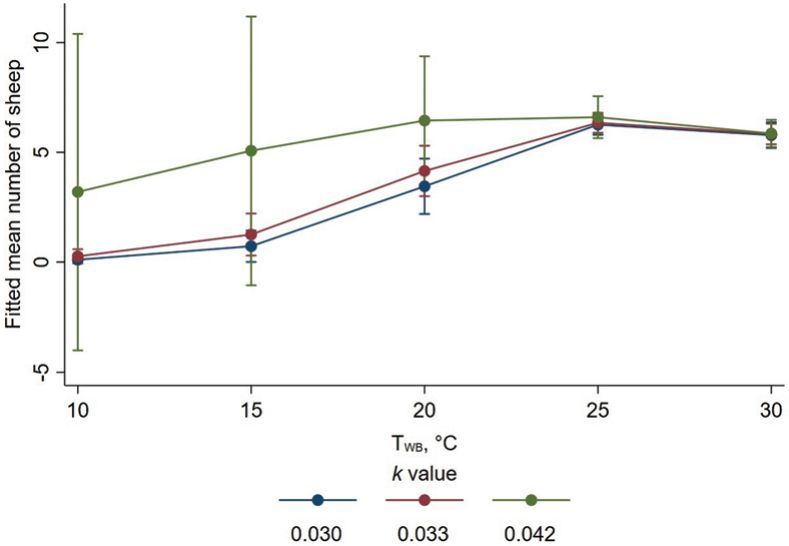
Fitted mean numbers of wethers that were standing as wet-bulb temperature (*T*_WB_) increased, for each allometric stocking density coefficient (*k*). Error bars represent 95% confidence intervals of fitted means. A zero-inflated negative binomial model was used. Fitted mean numbers account for the effects of *k* value and *T*_WB_ in both the count and inflate components of the model.

For number of wethers that were standing, considering the effects of the two parts of the zero-inflated model in combination, at low *T*_WB_ (i.e., 10 °C to 20 °C), the mean number of wethers standing was higher for higher *k* values (i.e., more floor space per wether). As *T*_WB_ increased, the number of wethers standing did not change appreciably at the higher *k* value (0.042). However, the number of wethers standing increased at the lower *k* values such that, at *T*_WB_ of 30 °C, numbers of wethers standing were similar over the range of *k* values studied (Figure 3).

**Figure 4.**
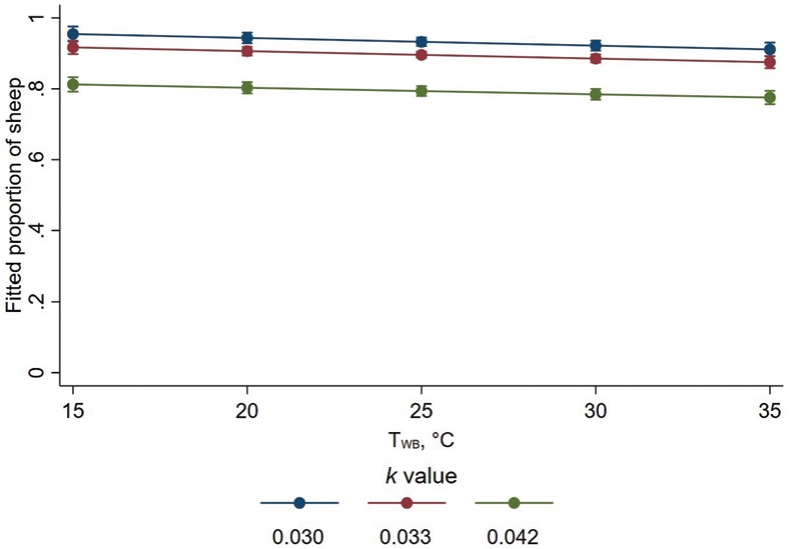
Fitted proportions of lying wethers that were in physical body contact with a conspecific as wet-bulb temperature (*T*_WB_) increased, for each allometric stocking density coefficient (*k*). Error bars represent 95% confidence intervals of predicted proportions. Fitted proportions were calculated as fitted numbers divided by the average number of lying wethers per pen whose body contact was known (11.74 wethers).

The proportion of lying wethers that were in contact with a conspecific decreased with more floor space per wether (i.e., with higher *k* value; Figure 4), decreasing by a factor of 0.87 for every 0.01 increase in *k* value (95 % CI 0.86 to 0.89; *P* < 0.001). Mean (± SD) proportions (across all time points) of lying wethers in physical body contact with a conspecific were 0.80 (±0.28), 0.78 (±0.28), and 0.66 (±0.29), for *k* values 0.030, 0.033, and 0.042, respectively. Increasing *T*_WB_ led to slight reductions in the proportion of wethers lying in physical body contact with a conspecific. The proportion was estimated as only changing by a factor of 0.977 for every 10 °C increase in *T*_WB_ (95 % CI 0.960 to 0.994; *P* = 0.009).

**Figure 5.**
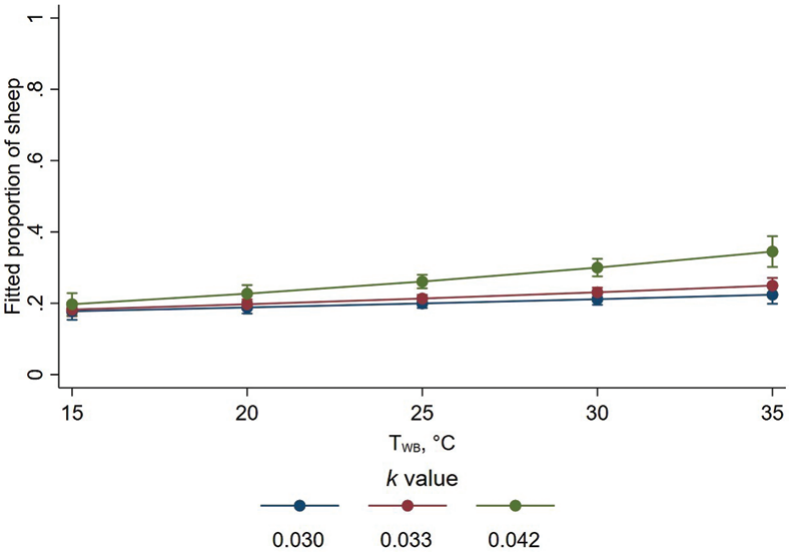
Interaction of wet bulb temperature (*T*_WB_) and allometric stocking density coefficient (*k*) on fitted proportions of lying wethers that had two or more outstretched legs. Error bars represent 95% confidence intervals of predicted proportions. Predicted proportions were calculated as fitted mean numbers divided by the average number of lying wethers per pen whose leg position was known (10.63 wethers).

Of wethers that were lying, there was some evidence that the proportion with two or more legs outstretched was determined by an interaction between *k* value and *T*_WB_ (*P* = 0.064; Figure 5), such that the proportion of the lying wethers that had two or more legs outstretched increased with increasing *k* value at higher wet-bulb temperatures, but this was not evident at lower wet-bulb temperatures. The estimated proportion of lying wethers with more than two outstretched legs increased by a factor of 1.01 (95% CI 0.79 to 1.31; *P* = 0.879), 1.17 (95% CI 1.03 to 1.32; *P* = 0.015), and 1.34 (95% CI 1.12 to 1.48; *P* < 0.001), for every 0.01 increase in *k* value at a *T*_WB_ of 10, 20 and 30 °C, respectively. Mean (± SD) proportions (across all time points) of lying wethers with more than two outstretched legs were 0.18 (±0.15), 0.21 (±0.16), and 0.23 (±0.18), for *k* values 0.030, 0.033, and 0.042, respectively.

*k* value had an effect on the proportion of lying wethers that were resting their head placed down (on the floor, themselves, or on a conspecific; Table 2). Of those lying, the proportion of wethers with their head down was less with more space, decreasing by a factor of 0.75 for every 0.01 increase in *k* value (95 % CI 0.72 to 0.79; *P* < 0.001). Mean (± SD) proportions (across all time points) of lying sheep that had their head placed down were 0.42 (±0.21), 0.41 (±0.20), and 0.30 (±0.18), for *k* values 0.030, 0.033, and 0.042, respectively. *T*_WB_ had a small effect on the proportion of lying wethers that had their head down. The proportion was estimated as only decreasing by a factor of 0.94 for every 10 °C increase in T_WB_ (95 % CI 0.90 to 0.97; *P* < 0.001).

Of wethers that were lying with their head down, the number resting their head on a conspecific decreased as space increased. The proportion of wethers resting their head on a conspecific was estimated as changing by a factor of 0.85 for every 0.01 increase (i.e., more space) in *k* value (95% CI 0.79 to 0.91; *P* < 0.001). Mean (± SD) proportions of wethers lying with their head down that had placed their head on a conspecific were 0.71 (± 0.25), 0.67 (± 0.25), and 0.55 (± 0.31), for *k* values of 0.030, 0.033, and 0.042, respectively. No important effect of *T*_WB_ was observed (estimated change in proportion for a 10 °C increase in *T*_WB_ = 1.02; 95% CI 0.98 to 1.05; *P* = 0.314).

*k* value had no important effect on the number of standing wethers with their head down (excluding those eating or drinking, estimated change in proportion for every 0.01 increase in *k* value = 1.07; 95% CI 0.97 to 1.18; *P* = 0.205). *T*_WB_ had some effect on the number of standing wethers with their head down. Of those standing, the proportion of wethers with their head down increased by a factor of 1.11 for every 10 °C increase in *T*_WB_ (95% CI 1.04 to 1.19; *P* = 0.003).

### Agonistic interactions

Mean (± SD) counts (across the behavior sampling days) of agonistic interactions for the 10-min observation periods were 31.5 (±20.6), 44.3 (±25.5), and 34.5 (± 17.7), for *k* values of 0.030, 0.033, and 0.042, respectively. Compared to wethers housed at a *k* value of 0.030, the mean number of agonistic interactions for wethers stocked at a *k* value of 0.033 was estimated as increasing by 12.8 occurrences (95 % CI 5.4 to 20.2; *P* = 0.001), but there was no evidence of a difference for wethers stocked at a *k* value of 0.042 (estimated difference 3.0; 95 % CI −4.40 to 10.40; *P* = 0.427). Compared to wethers housed at a *k* value of 0.033, the number of agonistic interactions for those housed at a *k* value of 0.042 were estimated as decreasing by 9.8 (95 % CI −17.18 to −2.39; *P* = 0.010). *T*_WB_ also had an effect on the number of agonistic interactions, with the estimated counts expected to decrease by 4.1 for every 1 °C increase in *T*_WB_ (95% CI 3.3 to 4.9; *P* < 0.001).

### Rumen temperatures

**Figure 6.**
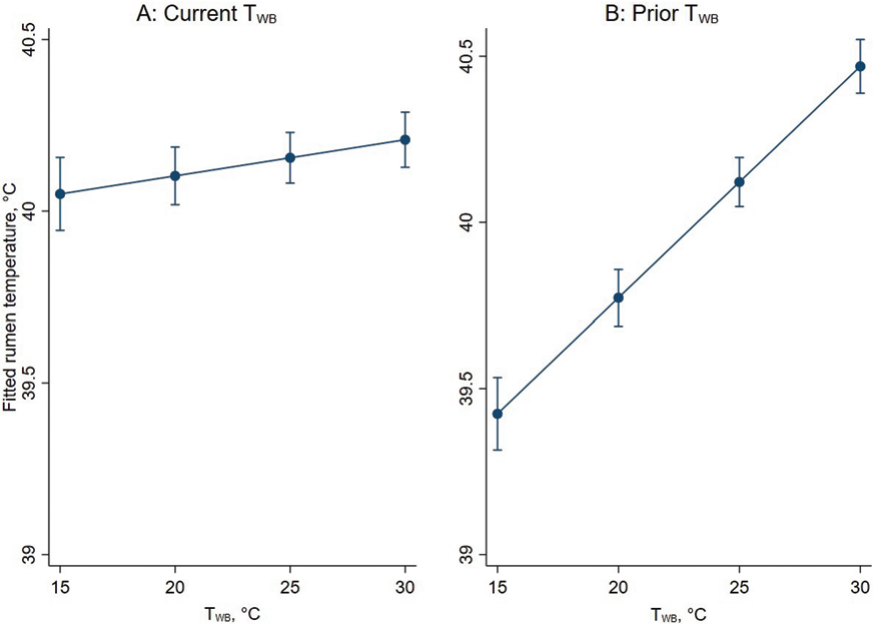
Fitted mean rumen temperature (°C) for changes in wet-bulb temperature (*T*_WB_) at the time of rumen temperature sampling (A) and for changes in mean *T*_WB_ from 1 to 6 h prior to rumen temperature sampling (B); effects of each are adjusted for the other. Error bars represent 95 % confidence intervals of fitted mean values.

Mean (± SD) rumen temperatures (across the duration of the study) were 40.2 °C (± 0.57), 40.0 °C (± 0.55), and 40.2 °C (± 0.53), respectively, for *k* values of 0.030, 0.033, and 0.042. The maximum observed *T*_RUM_ for these *k* values were 41.8 °C, 42.6 °C, and 41.6 °C, respectively. Mean (± SD) *T*_RUM_ for all wethers was 40.2 °C (± 0.55) across all days of the study, and 40.4 °C (± 0.45) from day 6 onwards. There was no important effect of *k* value on *T*_RUM_ (estimated change in mean for every 0.01 increase in *k* value = 0.05 °C; 95% CI −0.09 to 0.20 °C; *P* = 0.481). After accounting for mean *T*_WB_ for the 6-h prior to *T*_RUM_ measurement, a 1 °C increase in *T*_WB_ at the time of *T*_RUM_ measurement was estimated as causing a 0.01 °C increase in rumen temperature (95% CI 0.00 to 0.02 °C, *P* = 0.004; Figure 6A). After accounting for *T*_WB_ at the time of *T*_RUM_ measurement, a 1 °C increase in the mean *T*_WB_ for the 6-h prior to *T*_RUM_ measurement was estimated as causing a 0.07 °C increase in rumen temperature (95% CI 0.06 to 0.08 °C; *P* < 0.001; Figure 6B).

Some individual variation between wethers was evident, suggesting that some wethers had consistently higher temperatures and that rumen temperatures for some individuals increased more in response to higher *T*_WB_ at the time of *T*_RUM_ sampling. Results indicated some variation in the intercepts for the random effect of individual wethers (estimated standard deviation of intercept values after accounting for mean *T*_WB_ from 1 to 6-h prior to *T*_RUM_ sampling = 0.21; 95% CI 0.17 to 0.28). When the slope for *T*_WB_ at the time of *T*_RUM_ sampling was also allowed to be random, there was some variation in slope between individuals (estimated standard deviation of slopes after accounting for mean *T*_WB_ from 1 to 6-h prior to rumen temperature sampling = 0.007; 95% CI 0.005 to 0.01). This latter result indicates that a small proportion of wethers had slopes steeper than 0.024, calculated as 0.01 (the average estimate) plus 0.007 × 2 (two standard deviations above the mean). For a 10 °C increase in current *T*_WB_, the average wethers *T*_RUM_ increased by 0.10 °C, whereas for wethers with these steeper slopes, *T*_RUM_ increased by more than 0.24 °C. These differences between wethers are in addition to average effects of *T*_WB_ across sheep reported above.

**Figure 7.**
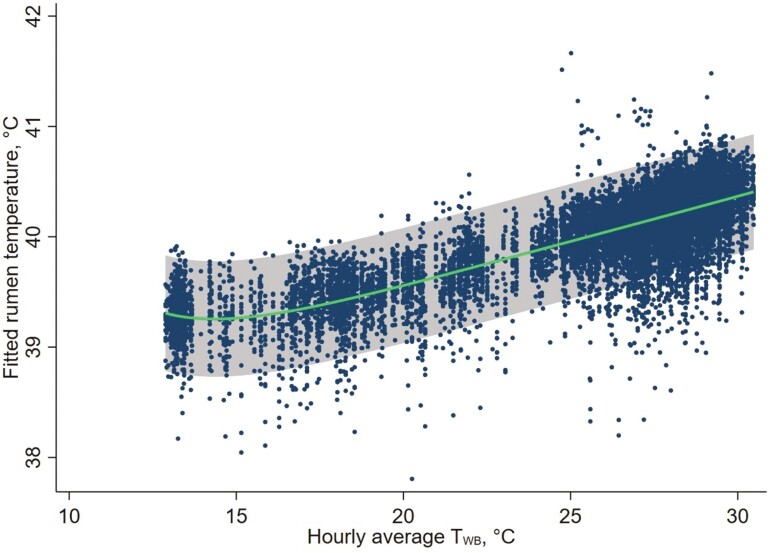
Three-dimensional fractional polynomial plot for the association between *T*_WB_ at the time of rumen temperature sampling and rumen temperature. The green line indicates the fitted fractional polynomial line and the grey zone indicates the 95% CI. Each blue dot represents a sheep-hour.

The relationship between *T*_RUM_ and *T*_WB_ at the time of *T*_RUM_ sampling appeared to be close to linear across the range of wet-bulb temperatures applied (Figure 7).

### Respiration rates

Mean (± SD) respiration rates (across all time points) were 171 (± 56.1), 167 (± 56.4), and 167 (± 48.4), for *k* value stocking densities of 0.030, 0.033, and 0.042, respectively. The respiration rates of the current study were comparable to those from research with similar climatic conditions (Lees et al., 2020b). For all *k* values collectively, the mean RR across all time points on days 1 and 3 (thermoneutral conditions), and from day 7 onwards (hot and humid conditions), were 66 (± 32.7) and 183 (± 40.4) bpm, respectively. The maximum observed RR for an individual wether during these same periods were 160 and 354 bpm, respectively. An interaction between *k* value and *T*_WB_ at the time of RR sampling was observed (*P* = 0.003). The estimated interaction term was −0.93 (95% CI −1.54 to −0.31), indicating that as *T*_WB_ increased, the effect of *k* value on RR decreased. At *T*_WB_ of 10 °C, for every 0.01 increase in *k* value, respiration rate was estimated as increasing by 12.4 bpm, whereas at *T*_WB_ of 30 °C, for every 0.01 increase in *k* value, RR was estimated as decreasing by 6.2 bpm.

### Liveweights

Mean (± SD) day 21 liveweights were 39.14 (± 4.17), 39.49 (± 5.30), and 38.41 (± 5.39) kg, for wethers housed at *k* value stocking densities of 0.030, 0.033, and 0.042, respectively. There was no important effect of stocking density on liveweights at day 21 (estimated difference between means for each 0.01 increase in *k* value = 0.02 kg, 95% CI −1.46 to 1.50 kg; *P* = 0.979). Unexpectedly, day 21 liveweights differed by room; after accounting for day −2 liveweight, wethers in room 2 were estimated to have a day 21 liveweight 2.1 kg less than wethers in room 1 (95% CI −3.6 to −0.6 kg; *P* = 0.006).

### Fecal glucocorticoid metabolite concentrations

There was no important effect of *k* value (estimated change in mean for every 0.01 increase in *k* value = −0.70 ng/g DM; 95% CI −2.82 to 1.43; *P* = 0.519) on the fecal glucocorticoid concentrations on days 7, 14, and 21 collectively (Table 4).

**Table 4.** Baseline FGCM concentrations (ng/g DM) and the means ± standard deviations of each wether’s change (*Δ*) at each sampling day relative to its baseline (ng/g DM). Results for each allometric stocking density coefficient (*k, n* = 72) are shown.

*k* value	Baseline	*Δ*7	*Δ*14	*Δ*21
0.030	13.95 (±2.39)	1.65 (±7.20)	3.96 (±7.26)	4.00 (±9.49)
0.033	15.57 (±5.35)	3.41 (±9.75)	0.03 (±4.21)	−0.23 (±7.30)
0.042	14.65 (±5.36)	0.45 (±7.44)	1.93 (±6.90)	2.47 (±7.42)

### Adrenal gland weights

Mean (± SD) day 24 adrenal gland weights for wethers housed at *k* value stocking densities of 0.030, 0.033, and 0.042 were 1.06 (± 0.47), 1.08 (± 0.43), and 1.01 (± 0.30) g, respectively. There was no important effect of stocking density on adrenal gland weights on day 24 (difference between means for each 0.01 increase in *k* value = −0.04 g, 95% CI −0.30 to 0.21 g; *P* = 0.736).

### Necropsy examination

There was no indication of ongoing respiratory distress in any of the carcasses. Fifteen wethers had parasitic cysts on their liver and two cases of pneumonia were identified, but scarring on the lungs indicated that these wethers had been affected by and recovered from pneumonia prior to the beginning of the experiment.

### Whole blood variables

There were no important effects of *k* value on day 21 whole blood variables (Table 5).

**Table 5.** Observed means, reference intervals, *P* values and estimated changes with allometric stocking density coefficient (*k* value) on day 21 whole blood variables.

Variable	Stocking density *k* value			Reference interval	Estimated change for a 0.01 increase in *k* value	95 % CI for estimated change	*P* value
	0.030	0.033	0.042				
Red blood cell count, ×10^9^ cells/L	10.72	11.36	11.09	8.2 to 12.3^1^	−0.11	−0.95 to 0.74	0.805
White blood cell count, ×10^9^ cells/L	6.99	6.57	6.60	5.0 to 14.0^1^	−0.40	−1.01 to 0.20	0.188
Lymphocyte count, ×10^9^ cells/L	3.17	3.01	3.14	2.0 to 5.7^1^	0.14	−0.42 to 0.69	0.623
Neutrophil count, ×10^9^ cells/L	3.36	2.89	3.11	1.5 to 8.6^1^	−0.37	−1.01 to 0.28	0.262
Neutrophil to lymphocyte ratio	1.20	0.96	1.13	0.1 to 1.2^2^	−0.06	−0.45 to 0.32	0.740
Packed cell volume, L/L	0.32	0.33	0.32	0.30 to 0.43^2^	−0.003	−0.02 to 0.02	0.780
Mean corpuscular volume, L/L	31.15	31.35	31.08	27 to 37^1^	0.41	−0.59 to 1.41	0.423
Hematocrit, L/L	0.33	0.36	0.34	0.28 to 0.39^2^	−0.003	−0.02 to 0.02	0.975
Hemoglobin, g/L	99.92	107.00	102.18	90 to 140^1^	−0.24	−7.06 to 6.58	0.946
Platelet count, ×10^9^ cells/L	500.33	430.55	494.82	260 to 1000^1^	31.20	−59.76 to 122.17	0.501

^1^Reference ranges reported from UC Davis Veterinary Medical Teaching Hospital (2001)

^2^Reference range reported from Lepherd et al. (2009).

## Discussion

This study was conducted to assess the welfare implications for sheep housed in three different allometric *k* value stocking densities while exposed to the climatic conditions that the Australian live export industry characterizes as the northern hemisphere shoulder period. Our results showed that reduced pen space did not impair indicators of sheep welfare for the experimental period and climatic conditions. This was demonstrated by the lack of important effects of *k* value stocking density on sheep hypothalamic-pituitary-adrenal (HPA) axis activity, adrenal gland weight, final LW, or whole blood parameters. However, some effects of *k* value stocking density on sheep behavior were observed. Some lying positions were prevented when stocking density was high (i.e., low *k* values); when provided with more floor space, more wethers adopted lying positions with outstretched legs, particularly when *T*_WB_ was high, and more wethers chose to lie in physical isolation from conspecifics, irrespective of climatic conditions. Importantly, *k* value had no important effect on the overall ability of wethers to lie, when judged by the number of wethers lying within a 24-h period or the synchronicity of lying. High *T*_WB_ led to increases in respiration rates, and there was an interaction between *k* value and wet-bulb temperature, such that wethers with more space had slightly lower RR at high wet-bulb temperatures. High *T*_WB_ led to increases in rumen temperatures, but no important effect of stocking density on this outcome was observed. Increases in RR and *T*_RUM_ suggest the wethers experienced heat stress, but the lack of effect on further aspects of biological functioning indicates that wethers were able to cope with the climatic conditions for 21-d, irrespective of their stocking densities, and that impacts on the expression of lying behaviors or physiological heat stress responses did not have negative consequences for biological functioning indicators of sheep welfare. Other factors which have the potential to impact sheep welfare during live export voyages were not present in this study; we purposefully limited the factors to pen space allowances under hot and humid conditions to gain an understanding of the role of stocking density under these conditions in isolation, and future work should aim to increase environmental complexity and provide information on the impact of other relevant factors.

Lying is a validated metric of animal behavior with regard to welfare, that has been applied in general contexts (Phillips and Petherick, 2015), and in specific relation to heat stress-related welfare concerns (Cook et al., 2007). For the wethers in the current experiment, stocking density had no important effect on the overall number of wethers lying within 24-h, and also had no important effect on the synchronicity of lying. The lack of effect on synchronous lying supports previous findings that *k* values as low as 0.027 facilitate synchronous lying in sheep housed under thermoneutral conditions (Australian Livestock Exporters’ Council, 2018; Mayes et al., 2022). With regards to the effect of climatic conditions, increased *T*_WB_ led to slight reductions in the number of wethers lying within 24-h. It is generally accepted that warm temperatures decrease the time animals spend lying (Galán et al., 2018), due to the increase in exposed surface area available for convective heat loss when animals are standing ­(Silanikove, 2000; Allen et al., 2015; Menchetti et al., 2021). In such environments, increased standing may inhibit an animal’s ability to achieve sufficient rest, and may compromise productive capacity through the diversion of energy (Moberg, 2000). However, the lack of effects on final LW suggests the reductions in number of wethers lying observed at high temperatures were not severe enough, or that the duration of the impacts on behavior was not long enough, to contribute to such effects.

Stocking density had no important effects on the overall expression of lying, but it did affect the body and head positions expressed by lying wethers. This may have further implications for quality of rest, an important consideration in the context of animal welfare (Temple et al., 2016). In the current study, the proportion of wethers lying with two or more outstretched legs increased by a factor of 1.17 for every 0.01 increase in *k* value, at a *T*_WB_ of 20 °C. At 30 °C *T*_WB_, the effect was increased so that the proportion of wethers lying with two or more outstretched legs increased by a factor of 1.34 for every 0.01 increase in *k* value. This indicates that sheep stretch out their legs at high temperatures when pen space permits them to do so. Similar results have been reported in sheep housed at different stocking densities under thermoneutral conditions (Mayes et al., 2022), and the interaction between stocking density and warm climatic conditions observed in the current study supports theories about the role of body surface area and heat exchange (Knowles et al., 1998). Sheep have highly vascularized skin under their flanks and may be seen attempting to spread their legs apart to increase heat loss by convection in situations where stocking density permits (Phillips, 2016); stretching their legs out while lying may achieve a similar outcome. We also found that when provided with more space, fewer wethers laid in body contact with conspecifics, and fewer wethers rested their head on a conspecific. These results further indicate a preference for some sheep to lie in physical isolation when available lying space permits them to do so, in agreement with previous research under thermoneutral conditions (Bøe et al., 2006; Andersen and Bøe, 2007; Mayes et al., 2022). We expected that this effect of stocking density would increase at high T_WB_ as wethers attempted to minimize heat transfer with other individuals. However, no important effect of *T*_WB_ was observed, suggesting that sheep prefer to lie in physical isolation from conspecifics irrespective of the prevailing climatic conditions.

When provided with additional space, the ability for more wethers to lie in physical isolation and with outstretched legs may facilitate additional passive heat loss (i.e., convection), due to increased available surface area for heat dissipation, and a reduced reliance on evaporative cooling through the respiratory tract. Often, the first reaction of animals exposed to hot environmental conditions is an increased respiration rate (Singh et al., 2016). Results from the current study indicate an interaction between stocking density and the *T*_WB_ at the time of RR sampling, such that respiration rates are slightly reduced for sheep provided with more space, at high *T*_WB_ (e.g., a reduction of approximately 6 bpm for each additional 0.01 in *k* value space allowance, at a *T*_WB_ of 30 °C). This may potentially be due to increased passive heat losses from available body surface area (Knowles et al., 1998), facilitated by lying in physical isolation and with outstretched legs. For comparison, the provision of shade for sheep housed outdoors at a dry-bulb temperature of 30 °C has been estimated to reduce RR by approximately 60 bpm (Marcone et al., 2021). While the study by Marcone et al. (2021) was conducted in a vastly different experimental setting (i.e., outdoors), their interpretation of the reduction in respiration rate remains relevant. These authors concluded that the shaded sheep (which had lower respiration rates) were less heat-stressed (Marcone et al., 2021). However, the ­reduction of only 6 bpm (for every 0.01 increase in *k* value space allowance) observed in the current study is not strong enough evidence to make a similar conclusion that sheep with more space was experiencing less heat stress. A small effect of *T*_WB_ in the 6 h leading up to RR sampling was also observed, as RR increased by 2 bpm for each 1 °C increase in the average *T*_WB_ across this time period. These effects of temperature are supported by the average RR of 183 bpm for all wethers from day 7 onwards, and an individual maximum RR of 354 bpm during this same period. A similar average RR of 182 bpm was reported during the last phase (day 24 to 29; daily mean dry-bulb temperature >30 °C) of a climate-controlled experiment conducted by Lees et al. (2020b). This RR, as well as the average and maximum RR observed in the current study, are substantially higher than a RR of 50 (Srikandakumar et al., 2003) or 75 (Lees et al., 2020b) bpm observed in sheep under thermoneutral conditions.

Core body temperature measures are the most validated and accepted method for detecting excessive heat load in animals, and the core temperature of sheep is generally maintained within a small range of temperatures, over a wide range of environmental conditions (Caulfield et al., 2014; Wijffels et al., 2021). This occurs as a result of various heat loss and gain mechanisms (Caulfield et al., 2014). Increases in respiration rates aim to prevent further increases in core temperature (Singh et al., 2016), but despite the interaction between *T*_WB_ and RR, the current study showed no effect of stocking density on *T*_RUM_, which is reflective of core body temperature. This suggests that more pen space did not facilitate additional heat loss.

With regard to the effect of climate, increasing *T*_WB_, at the time of *T*_RUM_ sampling and for the previous 6 h (similarly reported in cattle (Hahn and Mader, 1997)), did increase *T*_RUM_. Previous work has also reported increases in core temperature as a result of increasing environmental temperatures (Stockman, 2006; Stockman et al., 2011; Alhidary et al., 2012). Despite the increase in *T*_RUM_ observed, as a result of increasing *T*_WB_, wethers in the current study were able to maintain a normal core temperature on average. The normal core temperature of sheep under thermoneutral conditions is around 39.1 °C (Stockman et al., 2011), with an approximate range of 38.3 to 39.9 °C (Erickson et al., 2015). Across the entire study duration, the mean *T*_RUM_ for all wethers was 40.2 °C. In sheep, rumen temperature has been found to consistently exceed core temperature by 0.45 to 0.75 °C (Beatty et al., 2008). Adjusting the overall mean *T*_RUM_ observed in the current study by these values brings the mean to 39.5 to 39.8 °C, which is in the upper range of normal sheep core temperature (38.3 to 39.9 °C (Stockman et al., 2011; Erickson et al., 2015)). From day 6 onwards, THI values suggested sheep were at risk of experiencing extreme heat stress, and the mean *T*_RUM_ during this period was 40.4 °C. Adjusting this value to account for the digestive heat increment results in core temperature estimates of 39.7 to 40.0 °C; even when exposed to continuous heat and humidity, the average core temperature of the wethers was within the normal range, or slightly above it. This suggests the wethers were able to regulate their core temperature appropriately under the imposed climatic conditions, irrespective of stocking density. An increase in core temperature occurs when coping mechanisms, including increased respiration rates, become ineffective as a result of the heat load conditions (Wijffels et al., 2021). The fact that the wethers in the current study maintained a normal core temperature on average suggests that despite the high humidity and consequent reduction in effectiveness of evaporative heat loss through the respiratory tract (Berman, 2009), increased respiration rates remained an effective method of heat dissipation. Other research has found similar results; Merino wethers that were individually housed between 28 and 38 °C dry-bulb temperature for 7 d also exhibited increases in respiration rate, and were able to maintain a normal core temperature (Alhidary et al., 2012).

Even though the wethers in the current study were able to maintain a normal core temperature on average, a small proportion of wethers were less able to regulate their core temperature, and had higher increases in *T*_RUM_ relative to increased *T*_WB_. A maximum individual *T*_RUM_ of 42.6 °C was observed, and after adjusting for the heat increment associated with rumen fermentation (resulting in a range of 41.9 to 42.2 °C), this still exceeds the normal core temperature range for sheep by nearly 2 °C. Individual variation in the ability of sheep to tolerate hot and humid conditions has been consistently reported (Stockman et al., 2011; Menchetti et al., 2021), and our results provide additional evidence that while the majority of sheep may maintain a normal core temperature under hot and humid conditions, or when THI risk is considered extreme, others may be unable to effectively regulate their temperature, which may have negative effects on other aspects of physiology and welfare. Refining the assessment of heat stress during voyages is critical to identifying and managing the animals that are less able to cope with heat load. Of note, the level of individual variation in the ability to cope with heat stress will likely be greater in a commercial export environment, due to more variation in animal factors that contribute to this ability (i.e., age, genotype, and LW (Menchetti et al., 2021)).

Agonistic interactions are considered to indicate negative well-being (Dunston-Clarke et al., 2020), and wethers housed at a *k* value of 0.033 (i.e., the middle stocking density) performed more of these interactions compared to both other treatment groups. This is an unexpected result compared to previous research investigating similar stocking densities (Mayes et al., 2022). Wet-bulb temperature also affected agonistic interactions in the current study; the number of interactions decreased as *T*_WB_ increased. This contradicts previous findings of an increase in aggressive behaviors for lambs housed between 19 and 30 °C, compared to those housed between 12 and 18 °C (Menchetti et al., 2021). However, the reduction in agonistic interactions observed in the current study may indicate that wethers across all treatments were reducing their activity, in attempts to reduce heat production and eliminate additional heat load (Black et al., 1994; Menchetti et al., 2021). This is supported by observations made during sheep live export voyages, in which reduced activity has been observed in the mid to later stages of the journeys, when increased responses to warm climatic conditions were also observed (Willis et al., 2021). This reduction in aggressive activity at high *T*_WB_ suggests wethers were experiencing some degree of heat stress, and may have adapted their behavior to prevent additional metabolic heat production. However, high *T*_WB_ and THI values occurred later in the study and are resultantly confounded with time. As such, reductions in agonistic interactions may also indicate sheep adapting to their social environment (Houpt, 1998), despite being housed with the same individuals (in among a larger group of wethers) during adaptation.

The lack of important effects of *k* value stocking density on indicators of biological functioning (FCGM concentrations, liveweights, adrenal gland weights, and whole blood parameters) suggest that despite the behavioral effects observed, wethers did not perceive increased stocking density to be an ongoing stressor under the continuous hot and humid climatic conditions. Additionally, the level of heat stress suggested by the effects of *T*_WB_ on RR and *T*_RUM_ was not substantial enough to contribute to important effects on stress hormone production and biological functioning.

The use of adrenal gland weights for assessing heat stress in ruminants is not yet validated, but a reliable relationship between the duration and severity of prolonged stress and adrenal gland weights has been reported for some time (Sayers, 1950), which reflects the adrenal hyperplasia and hypertrophy that occurs in response to chronic stress (Ulrich-Lai et al., 2006). As HPA axis activity (i.e., FGCM concentrations) was unaffected by stocking density, it is reasonable to expect that the signs of adrenal hyperplasia or hypertrophy would also be lacking. Furthermore, while FGCM concentrations were generally higher than those reported in research investigating similar stocking densities under thermoneutral conditions (Mayes et al., 2022), the observed concentrations remained low compared to studies that have investigated the relationship between FGCM concentrations and pain (Bertulat et al., 2013; Stubsjøen et al., 2015) or 24-h road transport (Dalmau et al., 2014).

The effects of stress (Pascual-Alonso et al., 2017) and heat stress (Beatty, 2005; Alam et al., 2013) on leucocyte, lymphocyte, and neutrophil counts in ruminants are well documented. Stocking density had no effect on these parameters in the current study, supporting previous findings (Mayes et al., 2022). Additionally, all values fell within acceptable references ranges for sheep on d 21, suggesting that immune cell counts were also not altered by the climatic conditions. Heat stress has been found to affect other whole blood parameters, such as red blood cell counts, packed cell volume, and hemoglobin concentration (Srikandakumar et al., 2003; Alam et al., 2013; Sejian et al., 2013). Stocking density had no effect on these parameters in the current study, and all day 21 values fell within acceptable reference ranges for sheep, suggesting the level of heat stress experienced by wethers in all treatment groups was not substantial enough to contribute to changes in these biological functioning parameters. In other research where sheep physiology was largely unaffected by heat load conditions (Lees et al., 2020a), or where physiological variables were affected but returned to normal values rapidly (Stockman et al., 2011; Alhidary et al., 2012), this has been partly attributed to the ability of Merinos to tolerate hot and humid conditions.

To interpret the results appropriately, several important factors must be considered. First, stocking density has been consistently suggested as an important factor in determining how easily animals can dissipate heat (Knowles et al., 1998; Nielsen et al., 2022), but the stocking densities investigated in the current study are relatively similar; the additional space provided relative to the tightest stocking density may not have been sufficient to contribute to important changes in heat stress indicators (i.e., core temperature). In addition, while the climatic conditions mimicked real voyage climate data, unexpected temperature fluctuations during the shoulder period of the northern hemisphere summer may lead to higher wet-bulb temperatures than what was implemented in the current study, which may impose a larger heat stress risk. Similarly, adaptation period conditions for the current study were based on the average climatic conditions for the departure port, where sheep would be housed for at least 5 d prior to loading (Department of Agriculture Water and the Environment, 2021a). Inclement weather at departure ports may reduce the ability of sheep to later tolerate heat stress, through the skin thickening process that aims to restrict heat loss (Zhang and Phillips, 2019). Departure port temperatures have been found to be strongly associated with sheep mortality during voyages (Zhang and Phillips, 2019), so if departure port conditions are cooler than those imposed in the current study, the ability of sheep to tolerate heat stress during the voyage may be reduced. Also during voyages, animals kept on open decks may be exposed to radiant heat that was not considered in the current study (Carnovale and Phillips, 2020), and the removal of heat from closed decks depends on the effectiveness of forced ventilation systems (Collins et al., 2018). This becomes particularly important when stocking densities are higher, as temperature is increased above ambient levels as a result of metabolic head production (Carnovale and Phillips, 2020). Furthermore, temperature variations occur throughout live export vessels, and closed decks are likely to be warmer as a result of heat generated from the engine of the ship or other hot metal infrastructure (Collins et al., 2018; Carnovale and Phillips, 2020). If *T*_WB_ in some pens is increased as a result of these factors, adjustments in stocking density may become more important than what was observed in the current study. It is also important to consider that the experimental conditions were imposed for 21 d; sheep may experience additional stress relative to the *k* values or climatic conditions for intensive housing periods that persist beyond this, or when additional stressors are present. Additional factors that may induce stress during this mode of transport, such as movement associated with ocean swell, fecal pad development, human activity and noise, were not present. Also, due to the impact of ammonia on respiratory function, the build-up of this gas during voyages may limit heat dissipated from the respiratory tract (Caulfield et al., 2014). It is important to test how stocking density interacts with these other relevant stressors under hot and humid climatic conditions in future work.

## Conclusions

The aim of this study was to assess the welfare implications for sheep housed at three stocking densities, exposed to shoulder period climatic conditions of relevance to Australia’s live export industry. While we anticipated that reducing space allowance would lead to reduced sheep welfare, the various physiological and behavioral measures included in this study indicate that stocking density had limited important effects on sheep welfare or heat stress. Stocking density had an effect on some lying positions, suggesting that sheep were unable to lie in preferred positions when housed with less space (i.e., at low *k* values). Furthermore, the preference to lie with more than two outstretched legs was greater at high *T*_WB_, suggesting this lying position may facilitate additional heat loss and thermal comfort. We also provide evidence that the reliance on evaporative cooling through the respiratory tract may be reduced by providing more space at high *T*_WB_. Stocking density had no important effects on rumen temperature, and while increasing *T*_WB_ did lead to increases in rumen temperature, results indicated that most wethers could maintain rumen temperatures within the normal range. However, individual variation and maximum values suggested that not all wethers were able to effectively regulate their core temperature under these conditions, and discerning how to identify sheep that are less able to cope, particularly in the context of live export voyages, should be a priority of future research. We also provide evidence that pen space restriction and the climatic conditions were not ongoing stressors, reflected by the lack of changes in biological functioning. Sheep welfare was not impacted by the experimental *k* values or climatic conditions, based on the indicators of welfare and heat stress utilized in this study. This research has provided foundational knowledge regarding stocking densities for intensive housing and sea transport during periods of hot and humid climatic conditions, but the conclusions must be interpreted in the context of controlled experimental conditions. We suggest that it is possible to maintain an acceptable level of sheep welfare with the stocking densities investigated and high THI, under experimental conditions, but the challenge of identifying the factors present during live export voyages that cause welfare to fall below acceptable standards persists. As such, further research is required to determine the cumulative effects of environmental conditions during live export voyages (i.e., air ammonia build-up, and wave motion) and the relationships with thermoregulation and sheep welfare.

## Supplementary Material

skad223_suppl_Supplementary_Materials

## Abbreviations

CI: confidence interval
CP: crude protein
DM: dry matter
FGCM: faecal glucocorticoid metabolite
HPA: hypothalamic-pituitary-adrenal
LW: liveweight
ME: metabolisable energy
QASP: Queensland animal science precinct
RR: respiration rate
THI: temperature humidity index
*T*_A_: ambient temperature
*T*_RUM_: rumen temperature
*T*_WB_: wet-bulb temperature
