# Supplementary material for: The effects of stocking density on behavior and biological functioning of penned sheep under continuous heat load conditions
Source: J Anim Sci. 2023 Jul 1;101:skad223. doi: 10.1093/jas/skad223 (PMC10362936; doi:10.1093/jas/skad223)
Supplement: skad223_suppl_Supplementary_Materials [file skad223_suppl_supplementary_materials.docx]

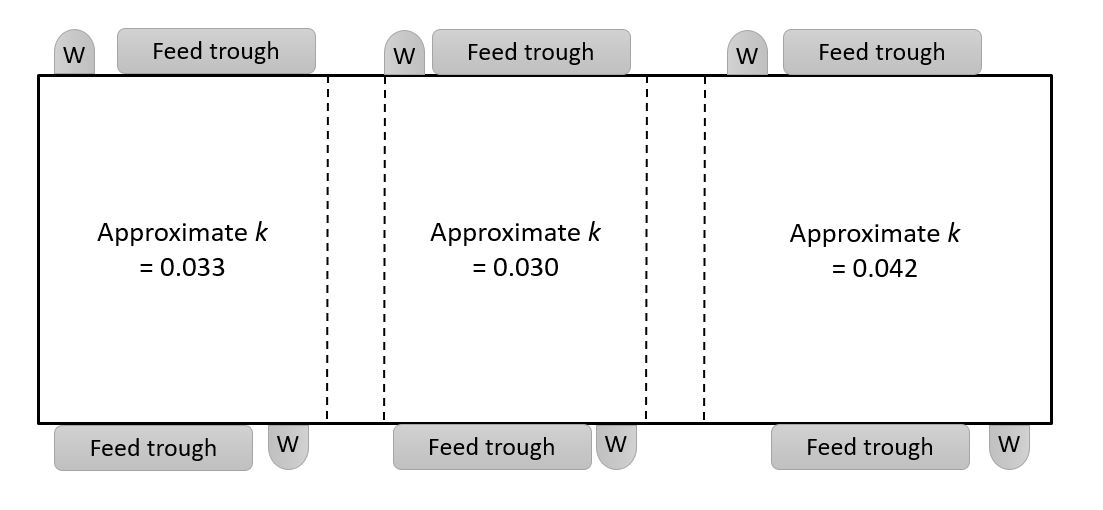


Supplementary Figure 1. Depiction of an adaptation pen (indicated by the solid black line) that could be divided into three treatment pens by inserting additional panels in the approximate location of the dotted lines, so that feed trough space and water troughs (W) were split equally across the three pens. Diagram not to scale.


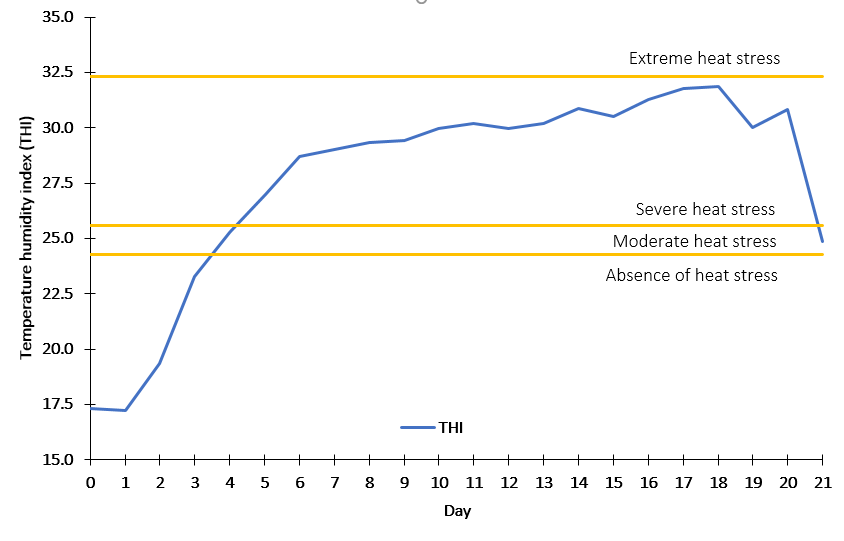


Supplementary Figure 2. Daily mean temperature humidity index across both climate-controlled rooms for each of d 0 (the last day of the adaptation period) to 21 (the last day of the experimental period). Yellow lines indicate heat stress risk limits as described by Lees et al. (2017).
